# Notes and Notices

**Published:** 1889-05

**Authors:** 


### NOTES AND NOTICES.

Died—Matthes.—Dr. G. Felix Matthes, of New Bedford, Mass., died March 17th, 1889. Dr. Matthes was one of our oldest and most successful practitioners; he graduated at Halle, Prussia, in 1836.

Removals.—Dr. L. R. P. Knox, from 1001 North Jefferson Avenue, to 3559 Olive Street, St. Louis. Dr. Charles S. Mack, from Boston to Fifty-seventh Street and Lake Avenue, Hyde Park, Illinois. Dr. F. M. Leitch has located at Campbell, Coles County, Illinois. Dr. L. L. Helt has located 284 S. Eighteenth Street, Columbus, Ohio.

Partnership.—Drs. W. Capps and S. E. Chapman have formed a co-partnership and located at Watsonville, California. Dr. Chapman was formerly at Forest Hill, California.

MARRIED.—Dr. L. L. Helt, now of Columbus, was recently married to Miss Frances E. Fenton, of Winchester, Ohio. Our best wishes for their success and future happiness.

SOUTHERN JOURNAL OF HOMŒOPATHY will hereafter be edited by Dr. G. G. Clifford and published at San Antonio, Texas. Dr. C. E. Fisher, who established the journal and has edited it for nearly six years, resigns the work on account of poor health, and will recuperate in Europe. Eventually Dr. Fisher expects to locate in California. THE HOMŒOPATHIC PHYSICIAN extends to the new editor a cordial greeting and best wishes for success in his new and difficult work.

A GOOD OPENING.—Dr. J. A. Hatzfield has removed from Hamburg to Pottstown, Pa. The Doctor writes that Hamburg offers a good opening for a physician who can speak both German and English, and who is "a *man* in the full sense of the word." Dr. Hatzfield offers to do all he can to aid such a successor. Address at Pottstown.

THE MEDICAL ANNUAL will be issued early in 1889 by E. B. Treat, publisher, 771 Broadway, New York. This will be the seventh annual issue of the English *Medical Annual*, a *resume* in dictionary form of new remedies and new treatment that have come to the knowledge of the medical profession throughout the world during 1888. The editorial staff will include articles or departments edited by Sir Morrell Mackenzie, M. D. (Laryngology), London; Jonathan Hutchinson, Jr., M. D. (Genito-Urinary Diseases), London; J. W. Taylor, M. D. (Gynæcology), Birmingham; William Lang, M. D. (Ophthalmologist), of London; James R. Leaming, M. D. (Heart and Lung), New York; Charles L. Dana, M. D. (Neurologist), New York; H. D. Chapin, M. D. (Pediatrics), of New York, and others, comprising a list of twenty-three collaborators, widely known in Europe and America. In its enlarged and widened sphere it will take the name of *The International Medical Annual*, and will be published in one octavo volume of about 600 pages at $2.75.

THE INTERNATIONAL HAHNEMANNIAN ASSOCIATION will meet at Toronto, June 18th–21st. Arrangements have been made for the accommodation of the members at "The Queen's," at rates of $2.50, $3.00, $3.50, and $4.00 per day, according to the location of rooms. The sessions will be held in the amphitheatre of the "Education Department," one of the chief objects of interest to visitors at Toronto, and easily accessible by horse-cars from the hotel. The first session will be held Tuesday, June 18th, at 10 A. M.

This early notice is given that members may have ample time to make arrangements for attending the meeting, and it is earnestly desired that there be a full attendance, not only on account of its influence in behalf of pure Homœopathy in Canada, but wherever Homœopathy is practiced. You are urgently requested to make every effort in your power to be present, to testify to the truths of the law of similars as seen in your daily practice, a duty that devolves upon every adherent of pure Hahnemannian Homœopathy.

S. A. KIMBALL, *Secretary I. H. A.*

THE JOURNAL OF HOMŒOPATHICS.—Dr. Harlyn Hitchcock has undertaken the publication of a new journal, with the above title, which is to be devoted to the teaching of the philosophy of Homœopathy. The subject certainly needs greater attention than it receives. We therefore wish Dr. Hitchcock all success in his arduous undertaking. The first number was issued in April. The journal is to be issued at No. 18 Broadway, New York, and subscription only *one dollar*. Success to you, Brother Hitchcock. There is always room at the top!

